# Combined nanofiltration and advanced oxidation processes with bifunctional carbon nanomembranes

**DOI:** 10.1039/d1ra01098k

**Published:** 2021-04-23

**Authors:** Barak Shapira, Tirupathi Rao Penki, Izaak Cohen, Yuval Elias, Raphael Dalpke, André Beyer, Armin Gölzhäuser, Eran Avraham, Doron Aurbach

**Affiliations:** Chemistry Department, Bar Ilan University Ramat Gan 592002 Israel eranchem@gmail.com; Institute of Nanotechnology & Advanced Materials, Bar Ilan University Ramat Gan 592002 Israel; Fakultät für Physik, Bielefeld University Universitätsstraße 25 Bielefeld D-33615 Germany

## Abstract

Wastewater reclamation is becoming a top global interest as population growth and rapid industrialization pose a major challenge that requires development of sustainable cost-effective technologies and strategies for wastewater treatment. Carbon nanomembranes (CNMs)—synthetic 2D carbon sheets—can be tailored chemically with specific surface functions and/or physically with nanopores of well-defined size as a strategy for multifunctional membrane design. Here, we explore a bifunctional design for combined secondary wastewater effluent treatment with dual action of membrane separation and advanced oxidation processes (AOP), exploiting dissolved oxygen. The bifunctional membrane consists of a CNM layer on top of a commercial ultrafiltration membrane (Microlon™) and a spray-coated reduced graphene oxide (rGO) thin film as the bottom layer. The CNM/support/rGO membrane was characterized by helium ion and atomic force microscopy, FTIR, XPS with a four-point conductivity probe, cyclic voltammetry, galvanostatic measurements, and impedance spectroscopy. Combined treatment of water by nanofiltration and AOP was demonstrated, employing a unique three electrode-dead end filtration setup that enables concurrent application of potential and pressure on the integrated membrane. For the model organic compound methylene blue, oxidation (by the Fenton reaction) was evaluated using UV-vis (610 nm). The rejection rate and permeability provided by the CNM layer were evaluated by dissolving polyethylene glycol (400 and 1000 Da) in the feed solution and applying pressure up to 1.5 bar. This demonstration of combined membrane separation and AOP using an integrated membrane opens up a new strategy for wastewater treatment.

## Abstract 2

A proof of concept of bi-functional membrane, for wastewater treatment, consisting of partially reduced graphene oxide and CNM is demonstrated.

## Graphical abstract

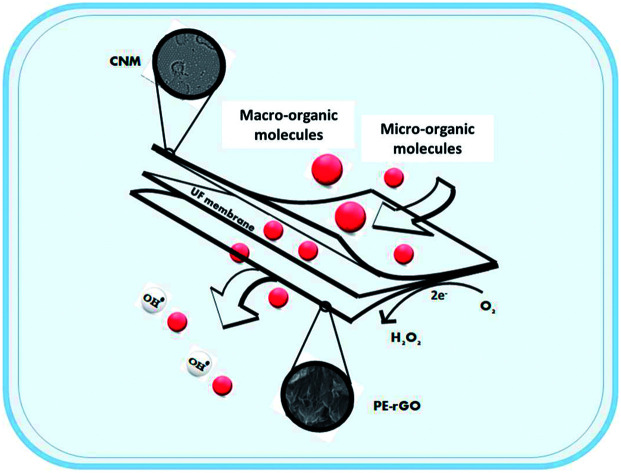

## 1. Introduction

Development of strategies toward municipal wastewater reclamation and reuse has been a main government objective in many countries, in particular those with areas with limited rainfall. Efficient reclamation policy not only reduces water shortage, but also helps conserve natural resources; recycled wastewater can be considered as a pseudo-sustainable source of water, as it is does not depend on rainfall, thereby increasing the reliability of the water supply.^1^ Some countries have set an ambitious goal—to meet up to 60% of the total water demand by development and assimilation of municipal and industrial wastewater reclamation technologies.

Municipal wastewater reclamation can be utilized in many fields, including agriculture (irrigation), industry (cooling towers), environment (streamflow augmentation), and urban (toilet flushing). Wastewater can also be reused for drinking water, depending on its composition and level of treatment.^2^ It contains a wide range of contaminants, ranging from suspended solids to the smallest inorganic salts. Traditional reclamation usually consists of a tertiary or even a quarterly treatment depending on the application. Common technologies for activated sludge effluents include adsorption,^3^ ion exchange membrane,^4^ and membrane bioreactor (MBR).^5–7^ The process also includes disinfection, commonly using chlorine. The choice of treatment method/s strongly depends on the quality required by the specific reuse application.

Integration of membrane separation technologies in wastewater treatment, applied mostly in tertiary and secondary effluents, and usually considered as polishing stages of municipal wastewater treatment, can play an important role in the effectiveness of the treatment. Whereas microfiltration (MF) is suitable for removal of large suspended solids, including larger micro-organisms and bacteria, ultrafiltration (UF) is suitable for removal of viruses and organic macromolecules down to around 20 nm. Removal of smaller organics and multivalent ions may be achieved by nanofiltration (NF).^6–8^

Micropollutants (MPs)—almost unavoidable in wastewater—can originate from natural or anthropogenic sources such as industry, agriculture, and domestic household, where sewage is known to be a major source.^5,6^ All have adverse health effects (in concentrations ranging from ppb to ppm) and pose a threat to ecosystems. MPs can be classified as disinfection by-products, pharmaceuticals, pesticides, and endocrine disrupting compounds, which interfere with the action of hormones in the endocrine system.^6^ Traditional wastewater treatment technologies are generally not effective in removing these MPs as many are hard to separate and resist biological degradation. Removal of MPs by membrane technologies was extensively investigated, especially with reverse osmosis (RO) and NF membranes,^9^ which can retain dissolved salts and solutes and are adequate for the majority of MPs that have molecular weight in the range of 200–400 Da.^7^ However, while these technologies provide a relatively effective barrier for almost all organic matter, two aspects are still a matter of concern. The first is the possibility of membrane fouling as a result of MP accumulation at the membrane surface, and the second is the unavoidable generation of secondary non-degradable waste, which needs to be managed.

Advanced oxidation processes (AOP) characterized by the generation of highly reactive, non-selective hydroxyl radicals offer a promising alternative to conventional treatment for removing organic pollutants.^10–13^ The ability of hydroxyl radicals to fully mineralize the micro-organic pollutants, or at least to produce less harmful intermediates, makes AOP an integral part of wastewater treatment. The most common process used to generate hydroxyl radicals is the combined use of catalytic oxidants such as ozone–ultraviolet, hydrogen peroxide–ultraviolet, and hydrogen peroxide–ozone.^12,13^ However, both membrane filtration processes and AOP are energy and chemical intensive, and thus incur significant cost. While membrane technology is energy intensive, AOP usually require consumption, transportation, and storage of chemicals such as hydrogen peroxides.

For membrane separation requiring less energy, the use of ultrathin porous membranes is favorable. In particular, 2D molecular materials like carbon nanomembranes (CNMs)^14–16^ are promising candidates, as separation can occur by sieving through molecular size exclusion. Due to their extreme “thinness” of 1–10 nm, CNMs show a permeation behavior characterized by extremely high water fluxes^12^ at low applied pressure, along with high ion rejection capability in the nanofiltration regime. Incorporation of CNMs in wastewater treatment processes could be the answer to the challenges posed by the accelerated pollution of water bodies associated with the growth in population and industry. In addition, the extremely thin active layer, with very low tortuosity and high pore density, exhibits very low ionic conductivity (toward monovalent ions), as shown here. This enables the design of an integrated three layered membrane, *i.e.* a CNM/support layer/thin film electrode material, which sustains an acceptable electric conductivity (both ionic and electronic) between two adjacent integrated membranes.

In this work, the idea of a bifunctional membrane is brought about. Gas permeation studies^17^ indicated that biphenyl CNMs transferred onto polydimethylsiloxane (PDMS) thin film composites reject molecules with kinetic diameters above ∼0.4 nm. CNMs with a thickness of about 1.5 nm were thus prepared from nitro-biphenyl-4-thiol (NBPT) precursors and transferred onto Microlon™ micro-filtration membranes in the first stage, providing a barrier for suspended solids. This was followed by thin film rGO coating on the back side of the membrane, providing the conductive matrix and the catalytic sites toward oxygen reduction and *in situ* hydrogen peroxide generation. The synergetic treatment of both membrane separation and AOP is demonstrated using a synthetic solution of suspended PEG particles and methylene blue (MB) as a model organic material.

## 2. Experimental section

### 2.1. Materials

Analytical-grade NaCl (>99%) and FeSO_4_·7H_2_O were purchased from Frutarom (Israel). Methylene blue (MB) solution, concentrated sulfuric acid (H_2_SO_4_, 98%), sodium nitrate (NaNO_3_, ≥99.9%), hydrogen peroxide (H_2_O_2_, 30% w/w in H_2_O), and hydrogen chloride (99.99%) were obtained from Sigma Aldrich™. Graphite KS-25 (Lonza), potassium permanganate (KMnO_4_, ≥99.9%, Bio-Lab Ltd) and double-distilled water (DDW) prepared in the laboratory were used.

### 2.2. CNM preparation

4′-Nitro-1,10-biphenyl-4-thiol (NBPT) self-assembled monolayers (SAMs) were prepared by immersion of a 300 nm Au (111) film on a mica support in dried, degassed dimethylformamide (DMF) solution. The Au (111) substrates were preliminarily UV–ozone cleaned. NBPT molecules were added to the solution, and the organic molecular monolayer was prepared during three days at room temperature (RT) under N_2_ atmosphere. After removal from the solution, the samples were rinsed with DMF and ethanol and dried with a nitrogen stream. The NBPT SAMs were then cross-linked by electron irradiation in high vacuum (<5 × 10^−7^ mbar) using a Specs FG20 flood gun with an energy of 100 eV and an applied electron dose of 50 mC cm^−2^. During this process, the nitro group of the NBPT SAM is reduced to an amino group due to dissociation of the hydrogen atoms from the phenyl rings. The cross-linked SAMs were transferred onto new substrates following a standard protocol.^18^ Polymethyl methacrylate (PMMA) was used as a transfer medium in order to preserve the integrity of the SAMs. The PMMA was evenly spin-coated on top of the cross-linked SAMs. The mica support was separated from the Au film by immersion in a KI/I_2_ etchant bath. Once the sample floated on a water surface for several minutes, it was released from the gold film by dissolution of the Au in the KI/I_2_ solution for 15 min. Finally, the CNMs were transferred onto a 140 μm thick polymeric support (3M Microlon™).

### 2.3. rGO preparation process

Partially exfoliated reduced graphene oxide (rGO) used for the bifunctional membrane that consisted of a CNM layer was prepared in two stages. In the first stage, GO precursor was prepared by a conventional modified Hummers method,^18^ followed by a second stage of thermal exfoliation to obtain a few layers of rGO. In brief, in the first stage, 184 ml of concentrated (98%) sulfuric acid solution contained in a conical flask was kept in an ice bath; to this solution, a 1 : 1 w/w ratio (∼4 g) of graphite and NaNO_3_ powders was added under constant stirring. After naturally reaching RT, 12 g of potassium permanganate were added slowly, and the mixture was continuously stirred for 2 h. To the above mixture, 184 ml of deionized water, followed by 18 ml of H_2_O_2_, were slowly added. The as-formed yellow-colored GO was filtered and washed several times with water, followed by 5% HCl and deionized water, until no sulfate ions were detected in the filtrate, and finally washed with ethanol; the obtained solid was dried in a vacuum oven for 24 h. Partial exfoliation of GO was carried out by fast exposure (thermal shock) of a small quantity in a pre-heated furnace (450 °C) under air atmosphere.^19^ After 5 to 10 s, the volume of the GO sample expanded several times, and the resultant few-layered partially exfoliated rGO was removed from the oven, subjected to further physical characterization, as described below, and used for the bifunctional membrane.

### 2.4. Incorporation of rGO in the integrated membrane

**Fig. 1:**
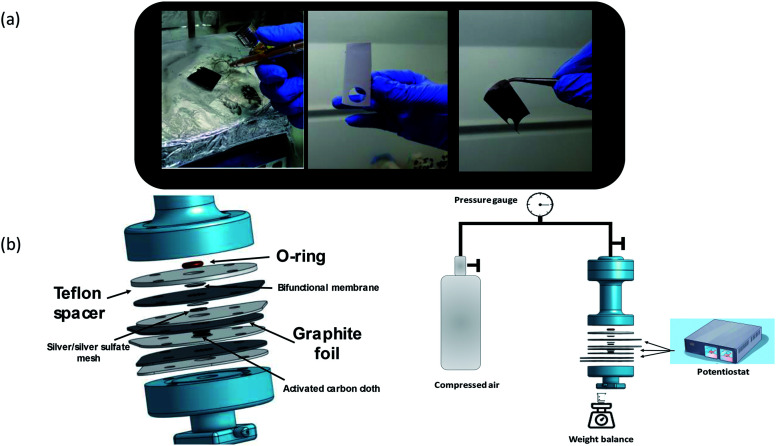
(a) From left to right—the spray-coating process, the CNM side of the membrane, and the rGO-coated side of the membrane. (b) The unique three electrode dead end filtration cell.

rGO (85 wt%), conductive carbon (acetylene black, 10 wt%) and polyvinylidene fluoride (PVDF, 5 wt%) were mixed well by grinding in a mortar–pestle. A few drops of *N*-methyl pyrrolidone (NMP) were added to form a dilute syrup. A thin film of rGO was obtained by prolonged spray-coating of the dilute slurry onto the back side of the Microlon™ support (see Fig. 1(a)), which was placed on a plate at a constant temperature of 120 °C. Prolonged coating, employing a dilute slurry, enables good adhesion of the rGO to the Microlon™ substrate and also between two adjacent spray-coated layers. The mass loading was continuously monitored until it reached a value of about 1 mg cm^−2^. The thickness of the layer was 0.1 microns. Pure water permeation tests (PWP, 35 LMH/bar, see eqn (1)) showed that the rGO coating did not affect the membrane permeability:1
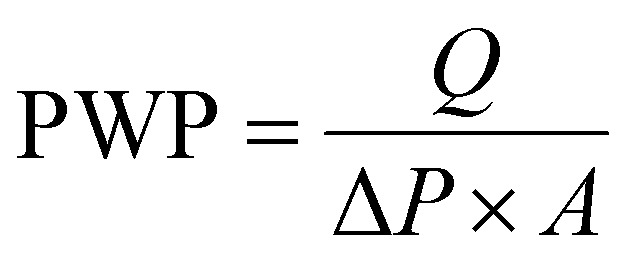
where *Q* is the water permeation volumetric flow rate (L h^−1^), *A* is the effective filtration area (m^2^), and Δ*P* is the trans-membrane pressure (bar).

### 2.5. Electrochemical measurements

Electrochemical measurements, namely cyclic voltammetry (CV) and constant current and voltage recording were conducted with a PGSTAT Autolab electrochemical measuring system from Eco Chemie, Inc (The Netherlands). All measurements were performed at room temperature (25 °C). For electrochemical analysis of rGO (CV and galvanostatic measurements), the water flux permeation was set to about 35 LMH.

### 2.6. Physical characterization of rGO powder

The as-prepared rGO was characterized by X-ray diffraction (XRD) with a Bruker Inc (Germany) AXS D8 ADVANCE diffractometer (reflection *q*–*q* geometry, 40 kV and 30 mA using a Cu Kα (*λ* = 1.5418 Å) radiation source, 0.2 mm receiving slit, and high-resolution energy-dispersive detector). The morphology of the samples was characterized by high-resolution scanning electron microscopy (HRSEM) with a FEI Megallen 400 L microscope and environmental scanning electron microscopy (ESEM) with an INSPECT-FEI instrument. Images of rGO were measured using a high-resolution transmission electron microscopy (HRTEM) JEOL 2100 microscope at an acceleration voltage of 200 kV. The specific surface area of the rGO powder was measured by the Brunauer–Emmett–Teller (BET) method using a Micrometrics Gemini 2375 analyzer with nitrogen adsorbate at 77 K. Raman spectra were measured in a back scattering configuration using a micro-Raman spectrometer (model HR 800, Jobin Yvon Horiba) with a He–Ne laser (excitation line 632.8 nm) and a microscope objective (50×, Olympus LWD).

### 2.7. Three electrode-dead end filtration system

Fig. 1(b) illustrates the specially designed three electrode-dead end filtration cell and electrochemical filtration system. Circular pieces (1 cm diameter) were cut from the membrane and mounted such that the CNM faces the pressurized side. Similar to a typical filtration cell, the membrane was installed into a 2 mm socket and pressed against a 2 mm O-ring. The effective area of the membrane was about 0.3 cm^2^, and the total volume of the cell was 15 ml (attached to a 500 ml dispensing vessel). The bottom part of the membrane was pressed against a perforated graphite foil serving as a current collector and also as a sealing gasket, as illustrated in Fig. 1(b). A Teflon spacer holding the same outer dimension as the graphite foil, with a 1 cm diameter hole in the middle, was mounted between a graphite foil and a subsequent graphite foil, also acting as a spacer, and played an important role in reducing the pressure on the cell components when the cell was sealed. The hole in the spacer was occupied with a 1 cm diameter separator (0.5 mm thick) and silver sulfate coated silver mesh as a reference electrode (RE), prepared as described previously.^13^ The reason for using this particular RE is explained in the following section. In the subsequent Teflon spacer, a 1 mm diameter activated carbon cloth (including a separator on top) was mounted. Porous carbon was chosen as the counter electrode (CE), mainly to avoid peroxide oxidation (the reverse reaction) and parasitic reactions due to extreme pH changes. That is also why the experiment was limited to 10 000 seconds. Prior to each experiment, the feed solution was pre-bubbled with pure oxygen for two minutes. The components are illustrated in Fig. 1.

Prior to filtration tests, the membrane was pre-compacted for several hours at a pressure of 2 bar with ultrapure water until a constant flux was obtained. Filtration tests took place under stirred conditions to minimize the effects of concentration polarization. Compressed air was used to pressurize the liquid above the membrane. Flux measurements were performed by weighing permeate at fixed time intervals on a top loading balance assuming a permeate density of 0.1 g ml^−1^. PEG retention was characterized based on gas chromatography-mass spectrometry (GCMS) measured in the filtrate and the retentate. The QTOF instrument (Agilent) used the following parameters: mobile phase A: water/0.1% formic acid, mobile phase B: acetonitrile/0.1% formic acid, ESI (positive), sheath gas flow 12, sheath gas temp (°C) 400, nebulizer (psig) 40, gas flow (l min^−1^) 8, gas temp (°C) 300, OctopoleRFPeak 750, Skimmer 1 45, fragmentor 180, nozzle voltage (V) 0, VCap 3500.

Analysis of MB degradation was measured using a UV spectrophotometer. The concentration of MB was determined based on a constructed calibration curve (1, 3, 5 and 10 ppm).

## 3. Results and discussion

### 3.1. Nanofiltration performance of the integrated membrane

**Fig. 2:**
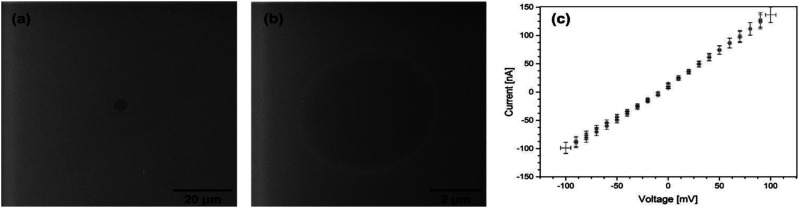
(a) and (b) HIM images of a NBPT CNM placed on a 5 μm diameter hole in a SiN membrane. (c) Current–voltage behavior in four-point measurement.

Owing to their unique properties, CNMs offer extraordinary water permeability and high impermeability even to solutes smaller than 0.6 nm, and may provide excellent water purification efficiency. In recent experiments, it was demonstrated that CNMs made of cross-linked terphenylthiol molecules are impermeable to ions.^14,15^ CNMs made of NBPT were thus first tested for their ion permeability. Fig. 2(a) and (b) shows helium ion micrographs (HIMs) of a NBPT CNM placed on top of a hole of 5 μm diameter in a SiN membrane that was then immersed in aqueous 0.03 M KCl solution. KCl has a neutral potential and does not deposit salt crystals on the membrane surface when used in low concentration, which makes it even more suitable for this experiment. Electrical measurements were acquired at RT with Ag/AgCl electrodes by using the four-point probe method, which records the current–voltage behavior; voltages between −100 and +100 mV were applied in steps of 10 mV, see Fig. 2(c).

The current–voltage curve for the NBPT sample reveals an ohmic behavior with an ion resistance of about 900 kΩ, of the same order of magnitude as reference measurements that yielded values of about 500 kΩ for samples with the same hole size (5 μm) without CNM. It was shown earlier that up to micron-sized defects most likely caused by the transfer process were observed in biphenyl membranes on PDMS.^17^ These imperfections may also provide paths for ionic currents. Nevertheless, the transfer process (as broadly elucidated in the experimental section) of the standalone CNM onto the Microlon™ support layer is a key point in successive fabrication of an “imperfection free” (or “defect free”) membrane. Such a CNM is a general description of a carbon based porous ultrathin membrane, regardless of its organic building precursors. The specific CNM along this work is denoted as an “NBPT-based CNM”. Nonetheless, a thorough study of the transport mechanism of solutes along the CNM and an in-depth examination of the relation between the transfer process, the precursors, and the fabrication process and membrane characteristics are subjects for further investigation.

The fabricated NBPT-based CNM membrane was initially mounted to measure the pure water permeability. Subsequently, the membrane was subjected to neutral solute and salt separation experiments through filtering of different solutions. The given solute rejection coefficient *R* (%) under the filtration process is given by eqn (2):2
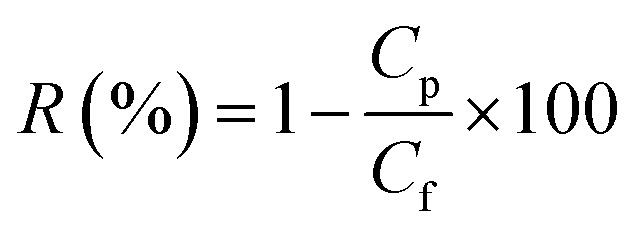
where *C*_p_ and *C*_f_ are the solute concentrations in the permeate and feed solutions, respectively.

**Fig. 3:**
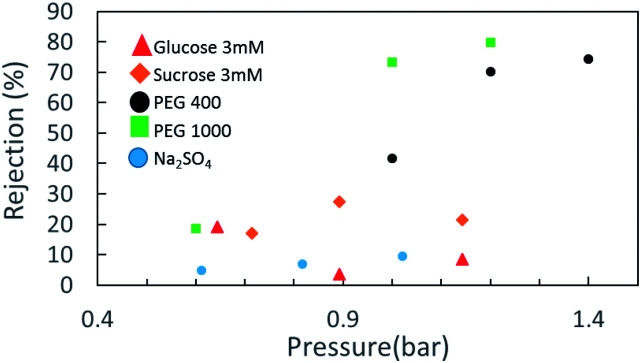
Solute rejection in NBPT-based CNM membrane of several materials.

Rejection tests with model salt electrolytes (mono and bivalent) and neutral solutes give a general representation of the membrane separation characteristics. Salt retention measurements with NaCl, Na_2_SO_4_ and MgSO_4_ (3 mM) and the monosaccharides glucose and sucrose (50 ppm) at a pressure difference of up to 1.5 bar were carried out with the three electrode-dead-end filtration cell in neutral pH. Fig. 3 indicates the retention behavior of the CNM membrane for the abovementioned solutes. As no significant rejection towards NaCl and MgSO_4_ was observed (not shown herein), and considering a slight rejection toward Na_2_SO_4_ and a moderate rejection toward glucose and sucrose, involvement of the Donnan exclusion mechanism and a static electrification characteristic of a negatively charged membrane cannot be ruled out in the separation mechanism, along with the size exclusion effect of the membrane.

However, when we move to neutral solutes with higher molecular weights—PEG 400 Da (particle size distribution *ca.* 5 nm) and 1000 Da (particle size distribution *ca.* 3.9 nm)—the rejection rates become much higher. The retention data for the NBPT-based CNM using a solution containing 50 mM PEG 400 shows that with increasing permeate flux from 20 LMH (∼0.6 bar) to 40 LMH (∼1.2 bar), the retention of PEG 400 increases substantially from less than 20% to 65%.

### 3.2. Physical characterization of rGO

The mechanism by which oxygen is reduced on carbon surface was extensively explored in the early 70's. As suggested by Yeager *et al.*,^21^ O_2_ reduction on carbon is involved in strong interactions of O_2_ with functional groups on the surface. This thesis was further supported by Tammeveski *et al.*, where quinone groups intentionally adsorbed to the basal plane of graphite were shown to give rise to oxygen reduction activity,^22^ As further evidence for the dominant role of functional oxygen surface groups, Evans and Kuwana^23^ showed that oxygen-containing groups can facilitate oxygen reduction by serving as mediators between the electrode and the electroactive species. In addition, surface oxidative treatments were shown to increase the coverage of quinonoid-type functional groups, making the carbon matrix more active toward oxygen reduction. Recently,^24^ it was shown that epoxy or ring ether groups in graphene located either on their basal planes or at plane edges play a major role in oxygen reduction catalyzation toward peroxide production with overpotential of less than 10 mV.

However, oxygen surface groups on standard carbonaceous materials are most likely to be found on the edge planes of the material. Graphene, owing to its layered structure, can endure oxygen functional surface groups not just on the edge planes, but the basal planes can be leveraged to bear an excessive amount of functional surface groups, thus making graphene a good substrate for oxygen surface groups. Epoxides and hydroxyl groups are most likely to exist at the basal planes, and a relatively low number of carbonyls, quinones, carboxylic acids, phenols, and lactones at the edges.^20^

Nevertheless, rGO aggregation as provided therein^25^ cannot be neglected when preparing rGO electrodes, as rGO can aggregate during the drying process, thereby substantially reducing the accessibility of the rGO basal planes to reactants (oxygen and water molecules in our case). The route by which GO is partially transferred into reduced GO, *i.e. via* low temperature thermal shock process might be an optimal compromise solution toward suitable resulted rGO in terms of the existence of catalytic sites, acceptable surface area and process scalability. It was shown before^16^ that graphene layers, after the transfer of GO into rGO could stack together to form a multi-layer “paper-like” structure where nanoscale pores generated, in a way that ions of electrolyte could easily get access to the gaps between the layers. On the other hand, the partial exfoliation preserves much of the original functional surface groups, or even can be the basis of the formation of new functional active sites.

**Fig. 4:**
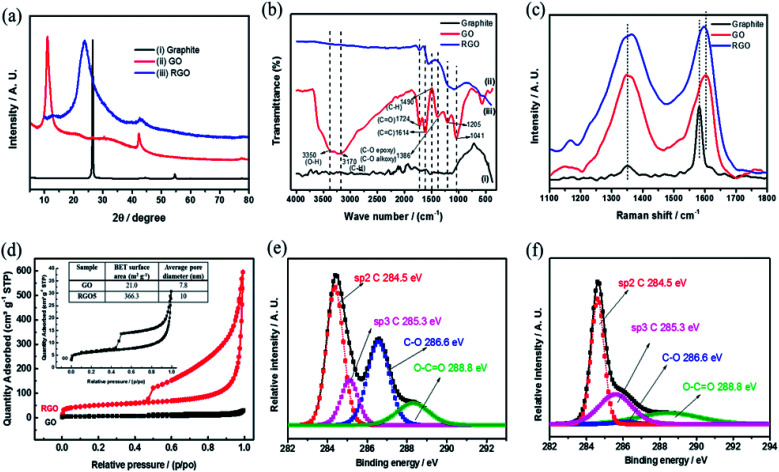
(a) XRD patterns, (b) FTIR spectra, (c) Raman spectra of graphite, GO and rGO (d) N_2_ adsorption–desorption isotherm of GO and rGO and XPS peak deconvolution of C (1s) core level of (e) GO, (f) rGO, respectively.

Fig. 4 includes a comprehensive physical characterization of the as prepared rGO powder compared with the source of graphite and graphite oxide. The rGO is characterized by XRD as shown in Fig. 4(a), curve (iii). The reflection peak (002) at 2*θ* = 26.5°, corresponding to of hexagonal graphite structure (Fig. 4(a) curve (i)), upon introducing the functional groups to the graphite forms graphite oxide (Fig. 4(a) curve (ii)), which is confirmed by the observation of a shift in the (002) reflection peak to 10.3°. This shift is due to the increase in inter layer distance. By the thermal reduction of the functional groups, the reflection peak (002) reflection reappears near to the 26.5° (Fig. 4(a) curve (iii)). The peak is in low intensity with broad reflection due to the disturbance in the regular stacking of graphite oxide that makes the diffraction peaks weak or even disappear. These XRD results confirm that the periodic layered structure of GO sheets was randomly exfoliated, leading to partial exfoliation by the thermal shock at 450 °C. The FTIR spectra of graphite, GO and rGO samples are shown in Fig. 4(b). As shown in the Fig. 4(b), curve (ii) confirms the reduction of graphite oxide to reduced graphene oxide by the disappearance of absorption bands at 3350 and 1724 cm^−1^ for O–H and C=O functional groups, respectively, which are clearly observed in graphite oxide (Fig. 4(b) curve (i)). The 1614 cm^−1^ band corresponds to C=C bond stretching.^20^ The peaks at 1205, 1041 and 1386 cm^−1^ represent stretching of C–O (epoxy), C–O (alkoxy) and deformation of O–H group, respectively, and all these functional groups are believed to exist, mainly, on the rGO basal planes. These groups were shown recently to be excellent active sites toward two electron reductions (ORR).^25^ Raman spectra (Fig. 4(c)) provide D and G bands at 1349 and 1601 cm^−1^ and are assigned to disorder and local defects. A shift of the G band from 1581 to 1605 cm^−1^, the intensity of the D band increased drastically for the rGO, when compared to the graphite.

The ratio (pristine graphite in comparison to rGO) intensities of D and G band (*I*_D_/*I*_G_) was dramatically increased, mainly due to the defects caused by the removal of carbon and oxygen during the thermal decomposition. The Raman spectra in conjunction to XRD results suggests a successful reduction of GO, resulting with highly defected rGO, attributed mainly to oxygen-containing defects and vacancies.

Fig. 4(d) presents the nitrogen adsorption–desorption isotherms and pore size distribution. The isotherms of rGO sample follows type IV isotherm implying on mesoporous structure with a surface area of 336.3 m^2^ g^−1^ (BET). According to Barrett–Joyner–Halenda (BJH) curves from desorption branch of isotherms, the pore size distribution is around 10 nm. The formation of rGO is further confirmed by XPS (Fig. 4(e) and (f)) of de-convoluted C1s spectra of rGO and graphite oxide by the Shirley background correction followed by Gaussian–Lorentzian curves fitting.

**Fig. 5:**
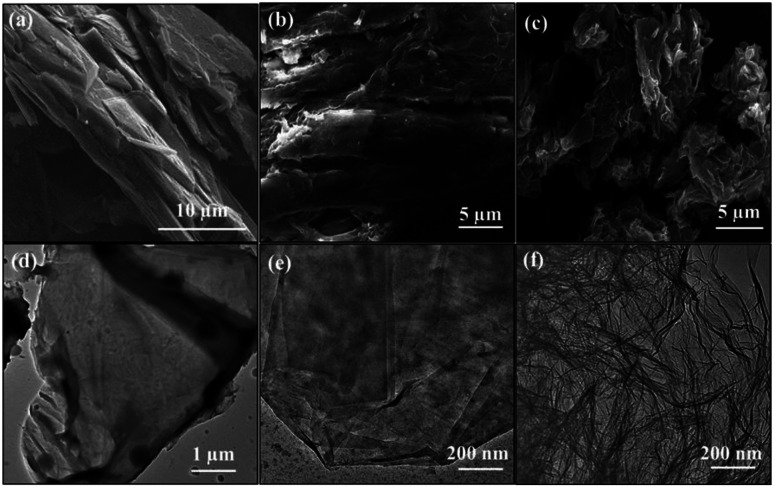
HRSEM and HRTEM images of (a) and (d) graphite, (b) and (e) GO and (c) and (f) GO, respectively.

The C1s de-convoluted spectrum for graphite oxide (Fig. 4(e)) reveals four characteristic peaks at 284.5, 285.3, 286.6 and 288.8 eV for sp^2^ hybridized C, sp^3^ hybridized C, epoxy, carboxyl and carbonyl groups, respectively. Fig. 4(f) shows that the sp^3^ C–C peak intensity decreases and the sp^2^C=C peak intensity increases, which confirms the reduction of functional groups upon thermal shock. In addition, HRSEM and HRTEM images of graphite, GO and rGO samples are shown in Fig. 5. Flaky morphology is observed for graphite (Fig. 5(a) and (d)) and GO (Fig. 5(b) and (e)). HRSEM imaging (Fig. 5(c)) and HRTEM (Fig. 5(f)) underline the multi-layer “paper-like” structure with a nano gap created between the layers after thermal exfoliation at 450 °C. Exfoliated samples of rGO exhibit a multilayered structure. When compared with graphite and GO, the as-formed rGO after thermal exfoliation reveals turbo-static arrangement of separated graphene sheets, as seen in Fig. 5(c) and (f).

### 3.3. Electrochemical characterization of the integrated membrane

Oxygen reduction can be followed by a direct four-electron pathway or by a two-electron (peroxide) pathway; the direct pathway in alkaline solutions is given by eqn (3):

3 O_2_ + H_2_O + 4e^−^ → 4OH^−^; *E*^0^ = 0.401 V

In acidic solution, oxygen reduction proceeds according to eqn (4)

4 O_2_ + 4H^+^ + 4e^−^ → 2H_2_O; *E*^0^ = 1.229 V

Note that if the pH is neutral, *E*^0^ = 0.81 V.

The peroxide pathway in alkaline solution is eqn (5)

5 O_2_ + H_2_O + 2e^−^ → HO^−^_2_ + OH^−^; *E*^0^ = −0.065 V

This reaction is followed by either reduction eqn (6)

6 HO^−^_2_ + H_2_O + 2e^−^ → 3OH^−^; *E*^0^ = 0.867 V

or decomposition *via* the disproportionation reaction eqn (7):

7 2HO_2_^−^ → 2OH^−^ + O_2_

In acidic solution, the peroxide pathway is eqn (8)

8 O_2_ + 2H^+^ + 2e^−^ + H_2_O_2_; *E*^0^ = 0.67 V

It is followed by either further reaction, or by the disproportionation eqn (9)

9 2H_2_O_2_ → 2H_2_O + O_2_

**Fig. 6:**
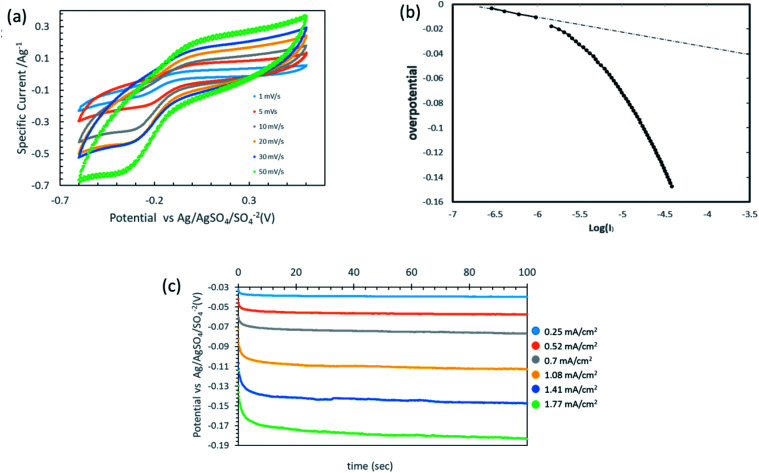
Electrochemical characterization of thin-coated rGO film. (a) Multiple cyclic voltammetry at different scan rates (1000 ppm Na_2_SO_4_). (b) Tafel curve of the thin film rGO followed by linear sweep voltammetry. (c) Potential time profiles at different current densities (1000 ppm Na_2_SO_4_, flux corresponds to 35 LMH).

Mostly, the oxygen-reduction reaction on the carbon electrodes proceeds predominantly as a two-electron process. Fig. 6(a) provides the cyclic voltammetry (CV) plots of the integrated membrane at different scan rates. As the output current in the CV plot is a linear combination of both faradaic and capacitive currents, the capacitive current needs to be excluded.^21^ As the background electrolyte is considered to be non-electroactive within the provided working potential window, and in the absence of faradaic reactions at anodic potentials, the current value in the anodic branch (the rectangular shape in the CV at the anodic branch) was established as the background current value and subtracted through the CV plot (see inset of Fig. 6(b)). The Tafel plot shown in Fig. 6(b) sets the onset potential of the reaction *versus* the RE as 0 V, compatible with a Tafel slope value of approximately 120 mV s^−1^ as reported by Yeager *et al.*

For carbonaceous materials, the oxygen reduction mechanism is suggested to be the one-electron transfer process as the rate determining step (eqn (10)), with a stoichiometric number of two for the overall electron (two electron pathway) as expected.

10 O_2_ → O_2(ads)_

The adsorbed super peroxide is believed to undergo the following reactions eqn (11) and (12):

11 O_2(ads)_ + e^−^ → [O_2(ads)_]^−^

12 2[O_2(ads)_]^−^ + H_2_O → O_2_ + HO^−^_2_ + OH^−^

Given that the micro-organic pollutants that are free to pass the membrane should react with the generated hydrogen peroxide until full decomposition, the rate at which hydrogen peroxide is generated must be correlated with the permeate flux. A simple linkage of the hydrogen peroxide (molar) concentration in the permeate flux, considering 100% conversion to H_2_O_2_, as a function of current density and permeate flux is given in eqn (13):13
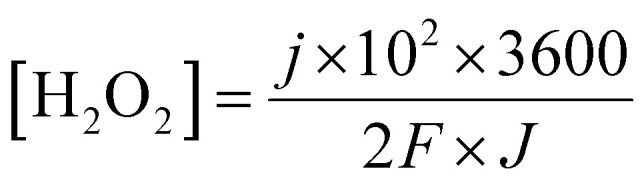
where *j* is the current density (mA cm^−2^), *F* is the Faraday constant, and *J* is the flux (in LMH).

Based on previous work on organic matter degradation using hydrogen peroxide^10,26^ the optimal ratio (in ppm) of H_2_O_2_ to organic matter should be in the range of about five to tenfold in favor of H_2_O_2_ (corresponds to about 5 mM). Considering an MP concentration of several ppm in the wastewater effluent and permeate flux in the range of 30–50 LMH, according to eqn (13) the membrane should cross a threshold of about 0.5 mA cm^−2^ with respect to the current density.

Fig. 6(c) presents the potential time profiles for different current densities. The membrane can sustain a relatively high current density, corresponding to about 2.5 ppm of H_2_O_2_ molecules per second for one square meter of membrane, yet with a low over-potential of less than 200 mV.

### 3.4. Combined nanofiltration and AOP

The concept of merging more than one functionality of a membrane is well established (the main function is usually solute separation). The design of multi-functional membranes is becoming an emerging topic in the last decade. Regarding integration of electrochemistry within the membrane structure, it is worth mentioning the extensive work by Jassby *et al.*^6,7^

In most cases, carbon nanotubes (CNTs) are incorporated within the membrane active layer (a crosslinking polymer, *e.g.* polyaniline or polyvinyl alcohol) to form a CNT/polymer composite, referred as an electroconductive membrane (EDM).^27^ EDMs were demonstrated to be effective at membrane separation processes including UF, NF and RO where the dispersed CNTs not only provide the membrane's electrical conductivity but also play a role in interesting electrochemical reactions such as oxygen reduction and chloride oxidation. Such membrane characteristics were utilized to prevent membrane fouling and scaling, and even for organic material oxidation.^6^

In this work, we adopt a different membrane design with a three layer membrane structure, considering that the transition from a lab scale electrochemical setup into a mature commercial prototype (or a module like the spiral wound module) may be involved with unexpected energy losses during operation, mainly an *iR* drop ascribed to electronic resistance of the electrode surface. In case the electrode material is embedded in the active layer, considering a spiral wound module for instance, the current should be collected from the membrane edges, unless incorporation of a stainless mesh pressing on the active layer surface is considered. Therefore, a non-integrated electrode layer could be disadvantageous in this regard.

Electrochemical oxidation of organic matter can be classified into direct and indirect oxidation. The latter takes place *via* a mediator, such as peroxides, while in direct electrochemical oxidation, organic compounds exchange electrons directly with the anode. However, direct anodic oxidation may sometimes lead to blockage of the anode. For example, phenol derivatives in water may undergo polymerization due to a chain reaction of phenoxy radicals intermediates. While a designated oxidation anode may undergo a self-recovery process, recovery of the integrated membrane, in case the membrane active layer is clogged, is questionable.

We believe that there is a trade-off between the two designs in terms of the durability of the membrane, the scalability of the setup, the projected operational and manufacturing costs, *etc.* A design with three layers, employing a conductive layer at the bottom, displaying filtration capabilities in the nanofiltration regime without compromising the ionic conductance across the membrane, poses a significant challenge.

**Fig. 7:**
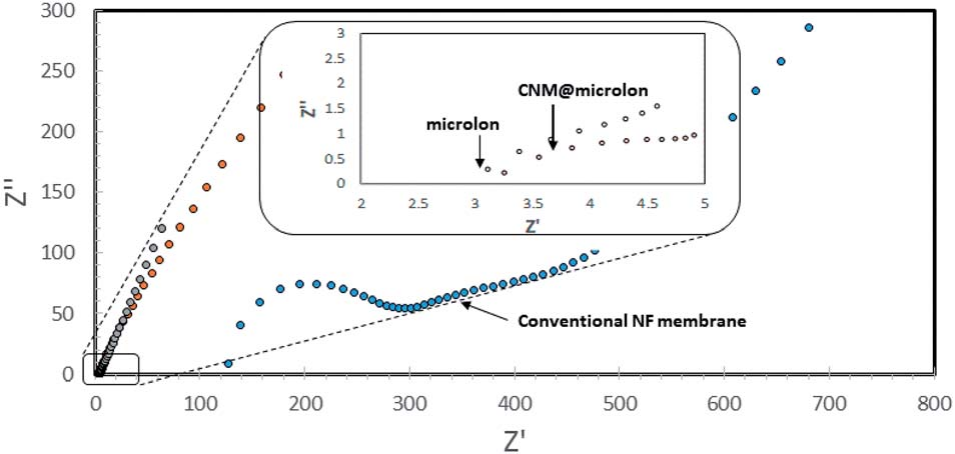
Impedance spectra of pristine and CNM-coated Microlon™ and commercial (Nadir) NF membranes with 1000 ppm NaCl in coin cell assembly.

Stressing the importance of the thickness of the CNM with this respect, a comparison between the ionic conductivity (1000 ppm NaCl) of pristine and NBPT-CNM coated Microlon™ was carried out. In brief, the investigated pre-wetted membrane was mounted between two stainless steel electrodes in a coin cell assembly such that the ionic conductance is governed by the membranes. As can be seen in the high frequency regime of the impedance spectra in Fig. 7 (the intercept with the horizontal axis), incorporation of CNM barely affects the ionic conductance of the pristine Microlon™; the ionic conductance of the CNM-coated Microlon™ membrane is more than tenfold higher compared to commercial NF membrane. Although the commercial membrane displays higher retention rates, the large difference in the ionic conductance is still appreciable.

As mentioned in the introduction, AOP are based on the generation of a hydroxyl radical, a nonselective oxidant with a high reaction potential (*E*^0^ = 2.8 V). The degradation sequence of the organic matter in water by hydroxyl radicals can be described by two mechanisms: abstraction of the hydrogen atom, *i.e.* alkanes or alcohols, to form water, or addition of hydroxyl radicals to olefins or aromatics for ring opening. However, in order to form hydroxyl radicals, the hydrogen peroxide should undergo a catalyzation reaction. Therefore, hydrogen peroxide is usually coupled with UV radiation as the energy source. Combining UV light with generation of hydrogen peroxide in order to eliminate MPs in the wastewater effluent could be the best strategy for improving the capacity of wastewater treatment plants. However, for demonstration of synergetic treatment of filtration and AOP, we applied the Fenton reaction^11,28,29^ to accomplish full degradation of the organic matter.

In brief, the interaction between ferrous ions and hydrogen peroxide and the oxidation of organic compounds can proceed by the following chain reaction eqn (14)–(17):

14 Fe^2+^ + H_2_O_2_ → Fe^3+^ + OH^−^ + OH*

15 Fe^2+^ + OH* → Fe^3+^ + OH^−^

16 RH + OH* → H_2_O + R*

17 R* + Fe^3+^ → R^+^ + Fe^2+^

where R represents the organic matter.

For demonstration of proof of concept, a synthetic solution containing 1000 ppm of Na_2_SO_4_ background electrolyte containing 50 ppm of PEG 1000 was admitted to the three-electrode filtration cell. The applied pressure (1.5 bar) was set to provide a permeate flux that corresponds to about 35 LMH. The current between the membrane and the CE was set to −0.25 mA (corresponds to about 0.9 mA cm^−2^).

Dosing of MB takes place immediately after the permeate comes out of the cell. Addition of MB as an MP model to the permeate collected out of the cell was done to eliminate false MB degradation reading due to MB adsorption to the porous carbon electrode, filtering by the membrane, or direct oxidation by the CE. Sodium sulfate was chosen as a background electrolyte to eliminate the possibility of chlorine formation on the CE and the subsequent hypochlorite compound that is also known to be a powerful oxidant.

After the collected permeate reached a certain level (several ml), it was dosed with FeSO_4_ solution, ending with a concentration of 2.5 ppm MB and 10 ppm Fe^2+^. The dosage of Fe^2+^ and MB was determined retrospectively after obtaining the H_2_O_2_ concentration in a parallel experiment (same conditions, 35 LMH, −0.25 mA), following the recommendations for Fenton's reagents dosage reported therein ref. 30. As expected, the integrated membrane exhibits good rejection toward PEG 1000 with 90% rejection rates.

**Fig. 8:**
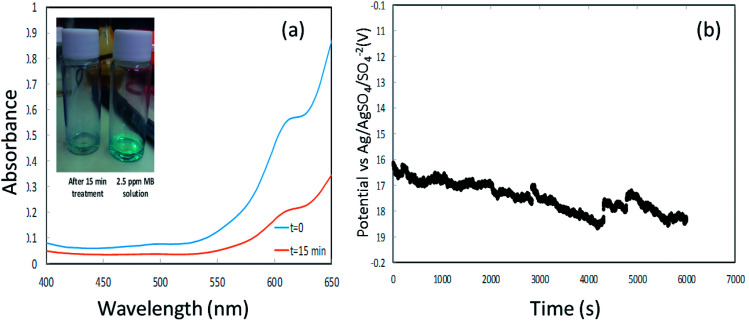
(a) is the UV spectra of methylene blue at *t* = 0 and after 15 min (inset shows the decoloration). (b) is the potential *vs.* time profile during galvanostatic measurement.

Fig. 8(b) shows the potential (*vs.* Ag/AgSO_4_/SO_4_^2−^ electrode) time profile along the galvanostatic operation. The potential seems to converge into a steady state and remains relatively low along the operation. The operation of such an integrated system with relatively low potentials not only projects the energy consumption of the process, which is of a great importance in wastewater treatment management, but also helps to avoid parasitic reactions and facilitates the sustained durability of the entire system, ranging from the membrane to the current collectors.

The envisaged concentration of H_2_O_2_ can be roughly calculated according to eqn (13) assuming 100% charge utilization. The H_2_O_2_ concentration determined by the titration method was 20 ppm, about 80% less than the expected value. We believe that this significant discrepancy is associated with reactions of H_2_O_2_ with the CE, which at this stage are not fully realized. However, the H_2_O_2_ concentration is sufficient for proceeding with the degradation process. Fig. 8(a) presents the UV spectra of MB at time *t* = 0 and after 15 min. A degradation of about 70% was accomplished (the inset shows the change in color). After 15 min, a plateau was reached in the MB concentration.

We present here a proof of concept for a synergetic treatment of nanofiltration and AOP in a single process with the design of a three-layered integrated membrane. The current design, with an NBPT-based CNM as the active layer, enables the rejection of macro-organic molecules (>4 nm) at relatively high fluxes and degradation of organic MPs that are free to pass the membrane.

## 4. Conclusions

A three-layer bifunctional NBPT-based CNM membrane for combined action of filtration and AOP utilizing dissolved oxygen in water was constructed and assembled in a three electrode-dead end filtration bench scale system. The integrated membrane showed a pore size cutoff value of about 5 nm and permeability of about 35 LMH/bar. Electrochemical measurements demonstrate the catalytic activity of the integrated membrane toward hydrogen peroxide generation with a low overpotential. A synergetic action of electrochemical oxidation by application of cathodic potentials to the integrated membrane concurrently with pressure application was demonstrated using a synthetic solution consisting of MB and PEG 1000. Degradation of MB (70%) and good rejection (>70%) of suspended particles (<5 nm) at pressures lower than 1.5 bar were obtained. The concept shown here opens up new strategies toward more efficient secondary wastewater effluent management and may contribute to mitigating the ubiquitous increase in the occurrence of micro- and bio-recalcitrant contaminants in water environments.

## Conflicts of interest

There are no conflicts to declare.

## Supplementary Material
